# Complete Genome Sequences of Low-Passage Virulent and High-Passage Avirulent Variants of Pathogenic *Leptospira interrogans* Serovar Manilae Strain UP-MMC-NIID, Originally Isolated from a Patient with Severe Leptospirosis, Determined Using PacBio Single-Molecule Real-Time Technology

**DOI:** 10.1128/genomeA.00882-15

**Published:** 2015-08-13

**Authors:** Kazuhito Satou, Makiko Shimoji, Hinako Tamotsu, Ayaka Juan, Noriko Ashimine, Misuzu Shinzato, Claudia Toma, Toshitsugu Nohara, Akino Shiroma, Kazuma Nakano, Kuniko Teruya, Yasunobu Terabayashi, Shun Ohki, Nobuo Koizumi, Shou Okano, Toshihiko Suzuki, Takashi Hirano

**Affiliations:** a Okinawa Institute of Advanced Sciences, Uruma, Okinawa, Japan; b Department of Molecular Bacteriology and Immunology, Graduate School of Medicine, University of the Ryukyus, Nishihara, Okinawa, Japan; c Department of Bacteriology I, National Institute of Infectious Diseases, Shinjuku, Tokyo, Japan; d Department of Biological Sciences, Okinawa Prefectural Institute of Health and Environment, Nanjo, Okinawa, Japan

## Abstract

Here, we report the complete genome sequences of low-passage virulent and high-passage avirulent variants of pathogenic *Leptospira interrogans* serovar Manilae strain UP-MMC-NIID, a major causative agent of leptospirosis. While there were no major differences between the genome sequences, the levels of base modifications were higher in the avirulent variant.

## GENOME ANNOUNCEMENT

*Leptospira interrogans* is a highly motile, obligate aerobic spirochaete that causes leptospirosis in both humans and animals, including wildlife, livestock, and pets (1). Leptospirosis is one of the most widespread (re)emerging zoonoses in the world, particularly in tropical and subtropical regions (2); it is both a neglected zoonotic disease (NZD) (3) and a neglected tropical disease (NTD) (4). *L. interrogans* colonizes the kidneys of reservoirs (e.g., rodents) and is shed in urine. Humans and animals become infected through environmental, occupational, or recreational activities involving contact with the urine or contaminated water or soil (5). Recently, in Okinawa, the southernmost prefecture in Japan, outbreaks have been reported among tourists enjoying water sports after heavy rainfall (6) and among American Marines attending jungle warfare training (7).

The pathogenic mechanisms of *L. interrogans* remain poorly understood; however, we have previously shown that virulence is correlated with increased phagocytic uptake and survival within macrophages (8) and two outer membrane proteins upregulated in the virulent variant enhance phagocytosis (9).

To date, complete genome sequences of *L. interrogans* are publicly available for five strains of four serovars (http://www.ncbi.nlm.nih.gov/genome/genomes/179). They consist of two circular chromosome genomes (around 4.3 Mb long with 35.0% G+C content and around 364.9 kb long with 35.0% G+C content) (10–15) and up to three plasmid genomes (around 64.1 kb long with 35.4% G+C content) (13, 14, 16). In this study, we performed whole-genome sequencing and *de novo* assembly of virulent and avirulent variants of *L. interrogans* to elucidate the pathogenomic mechanisms underlying leptospirosis.

*L. interrogans* serovar Manilae strain UP-MMC-NIID examined in this study had originally been isolated from the blood of a patient with severe leptospirosis (17, 18). The virulent and avirulent variants were derived by serial subculture after 1 (low) and 67 (high) passages, respectively (9). For each variant, extracted DNA was sheared to 20-kb fragments using g-TUBE (Covaris, Woburn, MA, USA) and converted into 20-kb template libraries. Whole-genome sequencing was carried out using the PacBio RS II platform (Pacific Biosciences, Menlo Park, CA, USA) with P5-C3 chemistry and 180-min movies. *De novo* assembly was conducted using the Hierarchical Genome Assembly Process (HGAP) (19) workflow involving consensus polishing with Quiver. This workflow constructed three single self-overlapping contigs representing two chromosomes and one plasmid for each variant. Annotation was added by the NCBI Prokaryotic Genome Annotation Pipeline (PGAP) (20). DNA base modification detection was also carried out using the kinetics data collected during the sequencing process. A summary of the genome statistics for both variants is shown in Table 1.

**TABLE 1:** Summary of the genome statistics for low-passage virulent and high-passage avirulent variants of pathogenic *L. interrogans* serovar Manilae strain UP-MMC-NIID

No. of passages	Variant type	Replicon name	Coverage depth (fold)	Genome size (bp)	G+C content (%)	No. of genes	No. of CDSs^a^	Accession no.
1 (low passage)	Virulent	Chromosome 1	501	4,238,972	35.00	3,562	3,431	CP011931
		Chromosome 2	413	358,378	34.91	298	294	CP011932
		Plasmid pLIMLP1	149	70,055	34.54	69	65	CP011933
67 (high passage)	Avirulent	Chromosome 1	533	4,238,922	35.00	3,561	3,422	CP011934
		Chromosome 2	405	358,377	34.91	300	295	CP011935
		Plasmid pLIMHP1	208	70,055	34.54	69	65	CP011936

a CDSs, coding sequences.

We found that while there were no major differences between the genome sequences, the levels of base modifications such as methylation were higher in the avirulent variant. These findings support the differential expression of hundreds of proteins between the strains demonstrated in our previous study (9). In addition, we discovered a novel plasmid in the pathogenic species.

### Nucleotide sequence accession numbers.

The complete genome sequences of both variants are available in DDBJ/ENA/GenBank under the accession numbers listed in Table 1.
